# SMART MOVE - a smartphone-based intervention to promote physical activity in primary care: study protocol for a randomized controlled trial

**DOI:** 10.1186/1745-6215-14-157

**Published:** 2013-05-29

**Authors:** Liam G Glynn, Patrick S Hayes, Monica Casey, Fergus Glynn, Alberto Alvarez-Iglesias, John Newell, Gearóid ÓLaighin, David Heaney, Andrew W Murphy

**Affiliations:** 1 Discipline of General Practice, School of Medicine, National University of Ireland, Galway, Ireland; 2 Graduate Entry Medical School, University of Limerick, Limerick, Ireland; 3 HRB Clinical Research Facility Galway, National University of Ireland, Galway, Ireland; 4 National Center for Biomedical Engineering and Science, National University of Ireland, Galway, Ireland; 5 Center for Rural Health, University of Aberdeen, Inverness, Scotland

**Keywords:** Smartphone, iPhone, Cell phone, Mobile phone, Exercise, Physical therapy, Application, Step counter, Pedometer and primary care

## Abstract

**Background:**

Sedentary lifestyles are now becoming a major concern for governments of developed and developing countries with physical inactivity related to increased all-cause mortality, lower quality of life, and increased risk of obesity, diabetes, hypertension and many other chronic diseases. The powerful onboard computing capacity of smartphones, along with the unique relationship individuals have with their mobile phones, suggests that mobile devices have the potential to influence behavior. However, no previous trials have been conducted using smartphone technology to promote physical activity. This project has the potential to provide robust evidence in this area of innovation. The aim of this study is to evaluate the effectiveness of a smartphone application as an intervention to promote physical activity in primary care.

**Methods/design:**

A two-group, parallel randomized controlled trial (RCT) with a main outcome measure of mean difference in daily step count between baseline and follow up over eight weeks. A minimum of 80 active android smartphone users over 16 years of age who are able to undertake moderate physical activity are randomly assigned to the intervention group (n = 40) or to a control group (n = 40) for an eight week period. After randomization, all participants will complete a baseline period of one week during which a baseline mean daily step count will be established. The intervention group will be instructed in the usability features of the smartphone application, will be encouraged to try to achieve 10,000 steps per day as an exercise goal and will be given an exercise promotion leaflet. The control group will be encouraged to try to walk an additional 30 minutes per day along with their normal activity (the equivalent of 10,000 steps) as an exercise goal and will be given an exercise promotion leaflet. The primary outcome is mean difference in daily step count between baseline and follow-up. Secondary outcomes are systolic and diastolic blood pressure, resting heart rate, mental health score using HADS and quality of life score using Euroqol. Randomization and allocation to the intervention and groups will be carried out by an independent researcher, ensuring the allocation sequence is concealed from the study researchers until the interventions are assigned. The primary analysis is based on mean daily step count, comparing the mean difference in daily step count between the baseline and the trial periods in the intervention and control groups at follow up.

**Trial registration:**

Current Controlled Trials ISRCTN99944116

## Background

Sedentary and physically inactive lifestyles are now becoming a major concern for governments of developed, and indeed, developing countries worldwide. Physical inactivity has been related to increased all-cause mortality, to lower quality of life, and to a higher risk of obesity, diabetes, hypertension, coronary heart disease, osteoporosis, fractures, colon cancer, breast cancer, prostate cancer, psychiatric disorders and an overall higher risk of hospitalization [1]. While the pathological effects and reductions in quality of life are of concern, so too are the associated economic costs for health services already working at capacity. According to the World Health Organisation, obesity rates have doubled since 1980 [2] and in addition, physical activity rates have fallen [3]. Of all types of physical activity, walking is the most commonly encouraged and most commonly reported form of leisure physical activity [4]. Evidence suggests that there are several requirements to successfully promote an increase in physical activity. Biofeedback is required on activity rates such as might be provided by a pedometer, realistic goal setting is required and lastly there must be motivation to undertake this change in behavior [4-6]. The powerful on-board computing ability of the smartphone has the ability to meet these requirements, with live feedback on activity measured as a step count as well as the ability to goal-set and provide a system of rewards for goals achieved. This system of live feedback is based on the use of accelerometers. Accelerometers are small single or uniaxial devices commonly found in all smartphones as well as electronics such as the Nintendo Wii controller and iPad (for example, they are the device responsible for rotating the screen from landscape to portrait). Accelerometers are an ideal choice for evaluating variability of movement and balance providing a non-invasive, portable method of measurement [7]. Pedometers containing accelerometers are well established as an acceptable form of monitoring and have been shown to be successful in promoting physical activity [8]. The advantage of using a smartphone with accelerometer-enabled application is that no additional piece of technology (that is, the pedometer) is required and people generally carry their mobile phone devices on their persons continuously. By carrying the smartphone throughout the day, with an appropriate application installed, physical activity is recorded and displayed as a daily step count. The saturation of smartphones within the mobile market (as of February 2012, 49% of US citizens had migrated to smartphones [9]), increases the potential benefit of, and ability to disseminate, using such persuasive technologies to monitor physical activity.

As far as the authors are aware after a systematic review of the literature [10], no previous trials have been conducted using a smartphone application to promote physical activity in any setting. Despite the rapid penetration of smartphones into the market place and the ready availability of multiple applications promoting physical activity, and indeed other health related outcomes, there remains little evidence for the effectiveness of such technologies. The results of a number of ongoing RCTs in the use of smart phone applications to promote health outcomes including the current trial, should begin to build an evidence base around the use of smartphone applications in the promotion of physical activity and the improvement of health related outcomes generally.

## Methods/design

### Setting

The study is taking place in the West of Ireland with recruitment in the community by the North Clare Primary Care Team (PCT). This is a rural based primary healthcare organization involving three family medicine centers and a population of approximately 8,000 patients. The study is coordinated by the Discipline of General Practice at the National University of Ireland, Galway, collaborating with the National Centre for Biomedical Engineering and Science and the Health Research Board Clinical Research Facility. All data collection, including the baseline screening and follow-up tests, occur in a community setting.

### Hypothesis

The formal null hypothesis to be tested in this study is that there is no change in mean daily step count between baseline and follow-up amongst participants in the intervention and control groups. The alternative hypothesis is that the intervention group using the smartphone application will show a significant increase in mean daily step count between baseline and follow-up in comparison to the control group.

### Sample size and power calculation

The sample size required for the proposed RCT is 80 participants. The sample size is based on the following assumptions. Assuming that the mean daily step count at baseline is 4,700 steps, it is estimated that over the length of the trial, the mean step count in the control arm will reduce by 470 (a 10% reduction) and increase by 940 (a 20% improvement) amongst those receiving the intervention [11]. Using a two-sample comparison of the mean improvement between the arms, a sample size of 33 subjects per arm is required to have 80% power at the 0.05 significance level to detect a difference in the mean improvement of 1,410 between the arms, with an estimated standard deviation of the improvement in step count of 2,000. To account for dropouts or loss of patients to follow-up, the sample size will be adjusted upward from 33 to 40 per group. All analyses will be conducted according to the intention-to-treat principle.

### Participants: recruitment and eligibility

**Figure 1:**
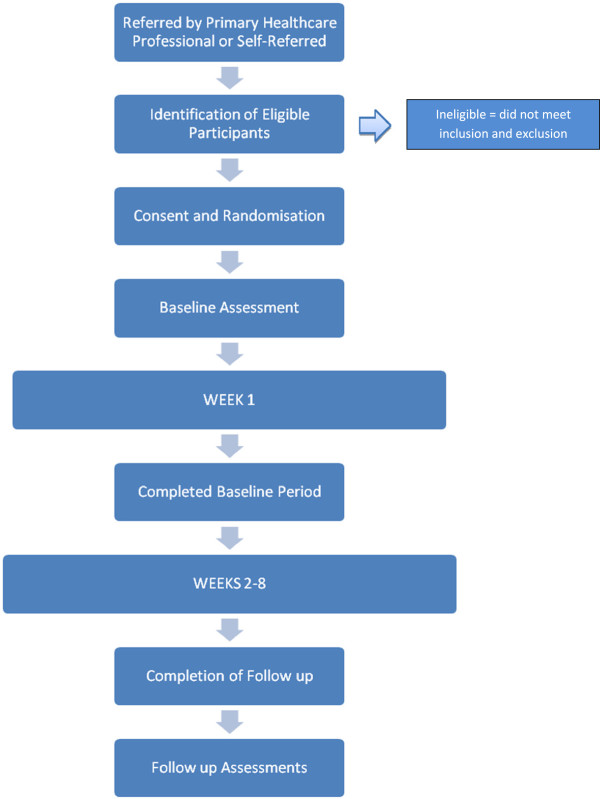
Participant flow through recruitment process.

Potential participants are referred to the trial team by their primary health care professional or they can self refer. Eligible participants are invited to attend a screening meeting with two members of the research team. A research engineer ensures that the potential participant has the appropriate mobile device (Android smartphone) and the ability to use it, and will record details of the technology being used and smartphone literacy. A research nurse ensures that the potential participant can undertake moderate exercise in the form of walking and then obtains informed consent from each participant. The research nurse then conducts the screening assessment by collecting relevant baseline clinical, anthropometric and psychological data as described in Table 1. After obtaining a written informed consent, they are randomized to intervention or control. The recruitment process is anticipated to span 12 months, from August 2012 to August 2013. The flow chart in Figure 1 represents the movement of a participant through the stages of recruitment to the study.

**Table 1:** Secondary outcomes: definitions and measurement techniques

**Secondary outcomes recorded at baseline and follow-up**	**Definitions and measurements**
Systolic blood pressure (SBP)	Mean difference in mean SBP between baseline and follow-up: the research nurse will be trained to measure blood pressure using the current British Hypertensive Society (BHS) guidelines. Specifically, the nurse will initially measure blood pressure in both arms, and then take the mean of three measurements from the arm with the highest initial diastolic reading. All measurements will be taken one minute apart after initial rest of five minutes. Blood pressure will be measured using an Omron I-C10 (HEM 7070) arm monitor if arm circumference is 17 inches or less or an Omron R7 (HEM 637) wrist monitor if arm circumference is greater than 17 inches. These devices have been independently validated [12].
Diastolic blood pressure (SBP)	Mean difference in mean DBP between baseline and follow-up: the research nurse will be trained to measure blood pressure using the current BHS guidelines. Specifically, the nurse will initially measure blood pressure in both arms, and then take the mean of three measurements from the arm with the highest initial diastolic reading. All measurements will be taken one minute apart after initial rest of five minutes. Blood pressure will be measured using an Omron I-C10 (HEM 7070) arm monitor if arm circumference is 17 inches or less or an Omron R7 (HEM 637) wrist monitor if arm circumference is greater than 17 inches. These devices have been independently validated [12].
Resting heart rate	Four readings will be recorded for resting heart rate from the automated devices used to measure blood pressure as described above. The first reading will be ignored and the mean resting heart rate will be calculated as mean of the last three readings.
Body mass index (BMI)	Height in cm and weight in kg will be recorded. BMI will be calculated as weight in kg/(height in cm) [2].
Psychological evaluation	a. Mental health score will be assessed using the HADS depression scale.
	b. Quality of life will be assessed using the EQ-5D and the EQ-VAS.

Eligibility for the study is determined using the following criteria:

Inclusion criteria:

participants are adults in the community with the following characteristics:

1. over the age of 16 years

2. active Android smartphone users

Exclusion criteria:

at the time of recruitment, individuals with the following characteristics are deemed ineligible to participate in the study:

1. non Android smartphone users

2. acute psychiatric illness

3. pregnant women

4. participants who cannot undertake moderate exercise

### Randomization process

The randomization process in this RCT uses random permuted blocks to ensure similar numbers of participants in the intervention and control groups.. In advance of participant recruitment, an independent researcher is responsible for generating the allocation sequence using the free computer  software program, http://www.randomization.com. The same independent researcher is responsible for assigning participants to the intervention and control groups. Thus, the allocation sequence is concealed from all study researchers until the interventions are assigned.

### Description of interventions/comparison groups

The study is a two-group, parallel randomized controlled trial. As such, following the screening visit, eligible participants are randomly assigned to the intervention and control groups in an equal ratio of 1:1. All participants have the smartphone application downloaded free of charge onto their smartphone but not made visible to the user. For the following week after the screening visit (baseline week), all participants are asked to carry their smartphone during walking hours and continue to operate at their normal physical activity levels. All participants are then contacted by SMS and mobile phone at the end of baseline week and asked to Email their baseline step count data to the research team using a ‘share data’ function within the smartphone application.

**Figure 2:**
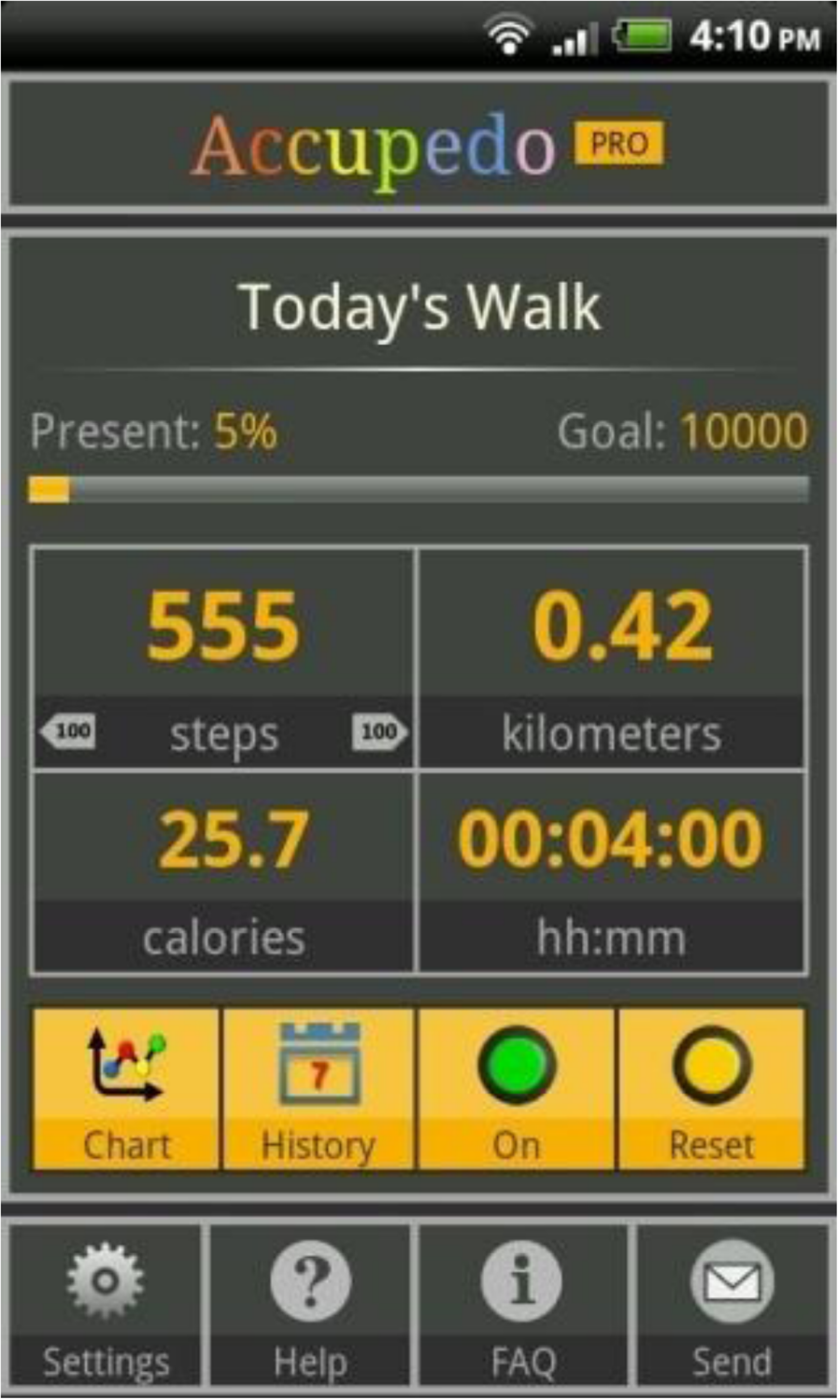
Home display page in smartphone application.

**Figure 3:**
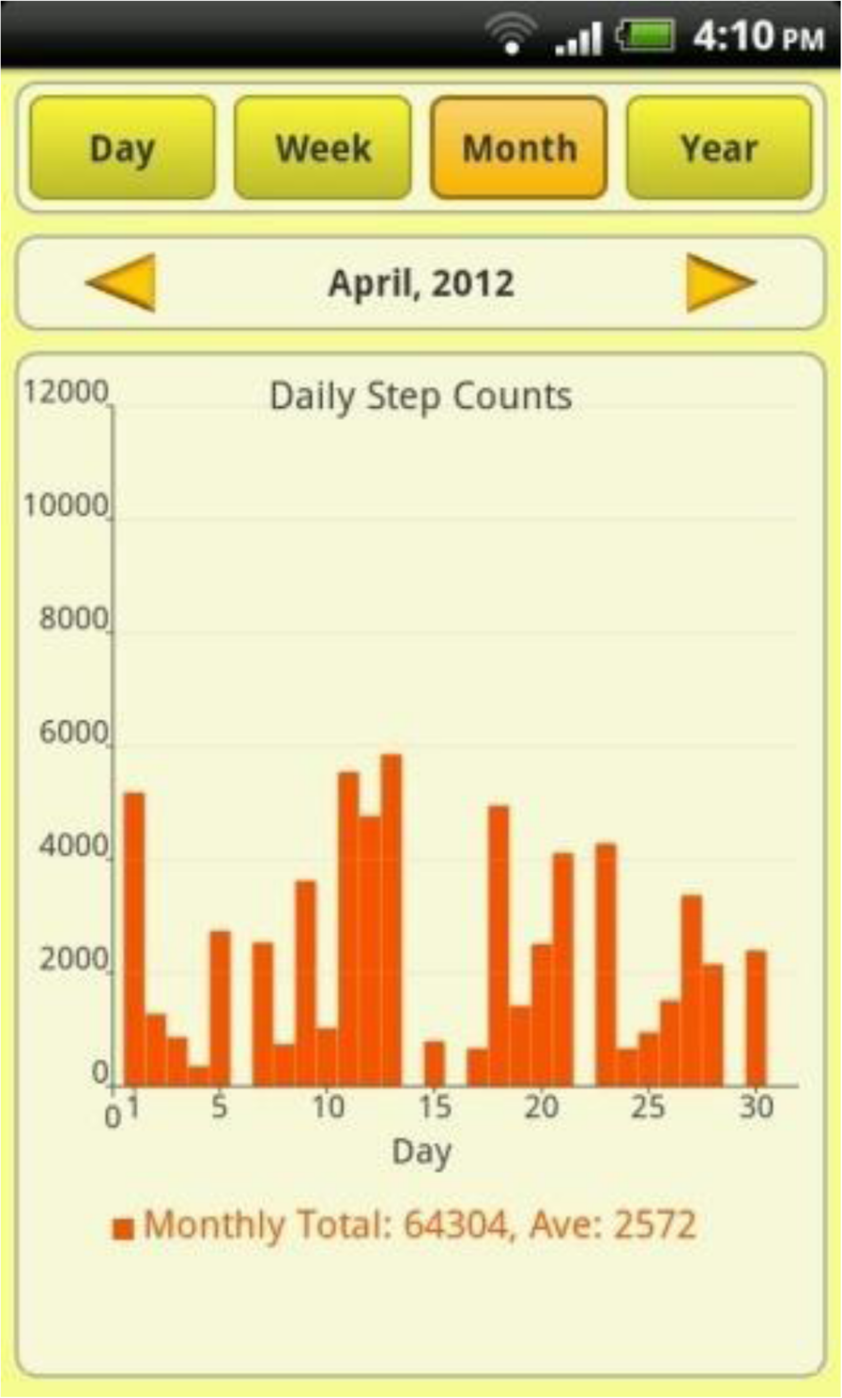
Chart display of daily step counts in smartphone application.

Once baseline week data collection is completed, those assigned to the intervention group are instructed on how to turn on the smartphone application display (Figure 2) and how to position the display widget on the smartphone home screen. Intervention group participants receive a short instruction by phone on the usability features of the smartphone application settings, feedback graphs (Figure 3), and continuous feedback on their daily step count. Intervention group participants are encouraged to interact with the smartphone application and are encouraged to try to achieve the physical activity goal of 10,000 steps per day. They are also issued with the Irish Heart Foundation ‘Be Active’ physical activity promotion brochure [13] by Email. Intervention group participants are then contacted after two weeks and at the end of the of the follow-up period by SMS and mobile phone, and asked to forward, using a ‘share data’ function within the smartphone application, all daily step count data up to that point. Finally they are instructed to keep their phone charged and to always carry it during walking hours.

Once baseline week data collection is completed, those assigned to the control group are encouraged to try to walk an additional 30 minutes per day along with their normal activity (the equivalent of 10,000 steps) [14] as an exercise goal. They are also issued with the Irish Heart Foundation ‘Be Active’ physical activity promotion brochure [13] by post or Email. Control group participants will then be contacted after two weeks and at the end of the of the follow-up period by SMS and mobile phone and are asked to forward, using a ‘share data’ function within the smartphone application, all daily step count data up to that point. Finally they are instructed to keep their phone charged and to always carry it during walking hours.

### Choice and description of the smartphone application

The team of researchers trialed various smartphone applications in an attempt to source a suitable autonomous, literacy-friendly and simple to use application, which possessed the key attributes that had already been identified in previous work [15], as important in applications which are designed to promote physical activity [16]. With evidence in literature on the benefits of using pedometers for promoting physical activity and walking interventions [4], researchers combined a list of potentially suitable iPhone and Android pedometer applications. As the Android operating system allowed the application to run continuously with or without interaction by the user it was decided to focus on the Android platform only. After pilot testing, the smartphone application Accupedo by Corusan [17] was chosen for study as it provided the most comprehensive list of advantages in applications of this type. These advantages include: easy to download; user-friendly; live feedback of step count to user; visually appealing graphic display of step count history with goal setting and goal achievement feedback; data sharing ability; inexpensive (once off purchase fee of €3.29 per user) and finally the application can run autonomously in the background, allowing researchers to monitor activity without user interaction or effort. At the time the trial began this smartphone application was only available on Android smartphone platforms. Accupdeo is now available on Android and iPhone platforms.

### Outcomes

The primary outcome variable is step count, measured daily for a week prior to treatment assignment (that is, baseline) and subsequently for a seven week follow-up period. Seven secondary outcomes will be measured at baseline and at the end of the follow-up period, namely: systolic blood pressure; diastolic blood pressure; resting heart rate; BMI; mental health as measured by HADS score; quality of life as measured by ED-5D and EQ-VAS. Table 1 outlines the definitions and measurement techniques for each of these secondary outcomes.

### Analysis plan

Daily step count (primary outcome variable) will be collected longitudinally as described above. All participants will also be invited back for follow-up testing (secondary outcome variables) within one week of finishing the trial. At this follow-up visit, to evaluate the effects of the trial on the secondary outcome measures, data on the same clinical, anthropometric and psychological variables measured at baseline (systolic blood pressure, diastolic blood pressure, resting heart rate, BMI, mental health as measured by HADS score, quality of life as measured by ED-5D and EQ-VAS) will be collected.

Baseline data will be compared across the two arms using suitable numerical summaries and graphical techniques. Functional data analysis techniques, using P-spline smoothers, will be used to graphically represent the eight week daily changes in step count in order to identify a suitable weekly summary statistic.

The effectiveness of the intervention will be assessed using a linear mixed model to compare the change in the primary outcome (within subjects and between treatments) when measured as the mean, median and total weekly steps while adjusting for the following covariates as appropriate: baseline step count, age, gender, socioeconomic status as measured by “medical card eligibility”, BMI, mean systolic blood pressure, mean diastolic blood pressure, mean resting heart rate, mental health as measured by HADS score, quality of life as measured by ED-5D and EQ-VAS and smartphone literacy as measured by whether the participant had activated Email on their smartphone. Those below a certain income level (for example, $238/€184 per week net income for a single person aged up to 65 years who is living alone or $261/€202 per week net income for a single person aged 66 years and over who is living alone) [18] are said to be ‘medical card eligible’ which allows access to family doctor or general practitioner (GP) services, community health services, dental services, prescription medicine costs, hospital care and a range of other benefits free of charge. Therefore, medical card eligibility provides socioeconomic data measured atan individual level. The selection of explanatory variables for inclusion in the final model will be based on a combination of clustering, tree-based methods and variable selection techniques.

It will be assumed that missing data are missing at random and therefore accounted for in the mixed model. The validity of this assumption will be investigated by looking at the missing data patterns and by modeling the probability of missing data based on the explanatory variables available. The sensitivity of the final conclusions to this assumption will be assessed using multiple imputations via chained equations and predictive mean matching assuming data are missing not at random. Model checking will be performed using suitable model diagnostics and residual plots. All statistical analyses will be performed using the software packages SPSS version 21.0, R version 2.14.3 and Minitab version 16.2.

### Qualitative analysis

Following the RCT, qualitative research will be undertaken to explore the perceptions, views and experiences of participants in the randomized controlled trial, comparing intervention and control groups. This work includes an exploration of the barriers and facilitators to making and maintaining lifestyle changes, as well as to the implementation of a physical activity program in the community generally. Toward this end, semi-structured, one-to-one interviews will be conducted with approximately 20 participants, from the intervention and control groups, within two months of completion of the trial. Participants will be purposively selected to ensure that a representative range of characteristics such as age, gender, socioeconomic status, baseline activity, BMI and smartphone literacy at the time of recruitment are taken into account. All interviews will be recorded and transcribed; data analysis will be based on themes derived from the transcripts. NVivo version 10, a qualitative research software package, will be used to facilitate qualitative data management and analysis.

### Ethical approval

Ethical approval was obtained from the Clinical Research Ethics Committee, Galway University Hospitals (reference number CA 760; date 14 August 2012).

## Trial status

Enrolment into the study started on 15 August 2012. Recruitment is expected to be complete by July 2013.

## Abbreviations

HADS: Hospital anxiety and depression scale; ISRCTN: International standard randomised controlled trial number; RCT: Randomised controlled trial; PCT: Primary care team; SBP: Systolic blood pressure; BHS: British hypertensive society; DBP: Diastolic blood pressure; BMI: Body mass index; EQ-5D: European quality of life-5 dimensions; EQ-VAS: European quality of life-visual analgoue scale; SMS: Short message service.

## Competing interests

The authors declare that they have no competing interests.

## Authors’ contributions

LG, PH, MC and FG are responsible for trial management, recruitment and data collection. LG and PH drafted the manuscript with contributions from all other authors. LG, PH, MC, FG, DH, GL and AM conceived and designed the study; JN and AA participated in the design of the study, and oversaw the sample size and power calculations, and developed the plan for statistical analysis of trial outcomes. LG revised and redrafted the manuscript, and gave final approval of the version to be published. All authors read and approved the final manuscript.

## Authors’ information

LG is a Senior Lecturer in General Practice at NUI Galway, Adjunct Senior Clinical Lecturer in the University of Limerick, and is Principal Investigator of this study; PH is an engineer and researcher in the Discipline of General Practice, NUI Galway and is technology lead for the trial; MC is a research nurse and trial manager and is currently working in the Discipline of General Practice, NUI Galway; FG is an implementation science researcher and Tutor at the Graduate Entry Medical School, University of Limerick; JN is a Senior Lecturer in Biostatistics in the HRB Clinical Research Facility at NUI Galway; AA is a post-doctoral statistics researcher in the HRB Clinical Research Facility at NUI Galway; GL is Head of Electrical and Electronic Engineering and Professor of Electronic Engineering, National University of Ireland, Galway; DH is Associate Director of the Centre for Rural Health, University of Aberdeen, Scotland, a health services researcher and is lead Principal Investigator on the ITTS project; AM is Professor of the Department of General Practice, National University of Ireland, Galway and General Practitioner Principal in Turloughmore, County Galway.
